# Supplementary material for: Long-lived giant circular Rydberg atoms at room temperature
Source: Nat Commun. 2026 Sep 15;17:9834. doi: 10.1038/s41467-026-77764-x (PMC13578691; doi:10.1038/s41467-026-77764-x)
Supplement: Supplementary file 1 [file 41467_2026_77764_MOESM1_ESM.pdf]

nature portfolio

Peer Review File

Long-lived giant circular Rydberg atoms at room temperature

Corresponding Author: Dr Florian Meinert

This file contains all reviewer reports in order by version, followed by all author rebuttals in order by version.

Version 0:

Reviewer comments:

Reviewer #1

(Remarks to the Author)
The manuscript under consideration investigates long-lived giant circular Rydberg atoms at room temperature. The two principal results are: (1) coherent control of individual circular Rydberg levels up to n = 103 with lifetimes exceeding 10 ms, and (2) optical tweezer trapping of Rydberg atoms on the ~100 ms timescale.

These are very impressive achievements. In particular, the work is a natural and important extension of the authors' previous paper, PHYSICAL REVIEW X 14, 021024 (2024), in which they demonstrated the generation of n = 79 circular states from low-l states. The present manuscript substantially extends this capability to higher principal quantum numbers and provides a systematic study of the lifetime enhancement for different circular states. The observed strong suppression of spontaneous emission, attributed to the Purcell effect induced by the ITO-coated windows, appears in very good agreement with the COMSOL simulation of the full structure. The demonstration of lifetimes exceeding 10 ms is, to my knowledge, the longest-lived Rydberg state experimentally demonstrated in a room-temperature setup. This is a noteworthy and significant result for the field.

Overall, the manuscript is clearly written, well structured, and experimentally thorough. I believe the work is suitable for publication in Nature Communications. However, before acceptance, I think a few important claims would benefit from clarification and further support.

Major comments

1. Clarification of claims regarding quantum information processing and simulation

In the third paragraph of the Introduction, the authors state:

"for comparison, our high n brings about a thousandfold stronger van der Waals blockade, and more than ten times larger dipole-exchange coupling. Combining these results forms the basis for establishing essentially dissipationless quantum simulation on the Rydberg platform on timescales two orders of magnitude longer than what is currently possible."

This statement feels somewhat too strong in its current form and may benefit from a more careful discussion.

A longer T1 is certainly helpful, especially in Rydberg-based platforms. However, it is not yet clear that the strong blockade of circular Rydberg states can be directly exploited in a practical gate protocol, since excitation first starts from low-l states and then requires an adiabatic RF or microwave transfer to the final circular state. As far as I know, there is no direct blockade-gate scheme that uses the circular-state blockade in the same way as standard low-l Rydberg gates.

In fact, previous proposals such as PRX Quantum 2, 030322 (2021) implement gates through coherent Sz_i Sz_j-type interactions, where the relevant figure of merit is not only T1, but also T2. Similarly, for quantum simulation applications, the coherence time can be limited by several other effects, including atomic motion and electric-field stability. The latter may become even more important at higher n because of the stronger polarizability of the circular states. In addition, for circular states, interaction-induced atomic motion has recently been discussed in PRX Quantum 6, 010353 (2025), which suggests that motional effects may also become relevant in the coherent dynamics.

For these reasons, the demonstrated enhancement of T1 does not necessarily imply a proportional improvement in coherent

many-body operations or gate quality. I think the manuscript would be stronger if the authors could either soften this claim or provide additional discussion to better justify it. For example, it would be helpful to comment on the expected T2 limitations in the present platform, or to outline what technical developments would be needed for the long T1 to translate into an actual quantum information or quantum simulation advantage. A short discussion of possible future improvements, such as better electric-field control, reduced motional effects, or protocols specifically designed to exploit the circular-state manifold, would make this part much more convincing.

2. State preparation fidelity should be discussed more clearly

I also think it is important to state the fidelity of the Landau-Zener preparation process more explicitly.

In the previous paper, PHYSICAL REVIEW X 14, 021024 (2024), the fidelity to |79C> was already around the 70% level. In the present work, the current Landau-Zener process to reach higher n would seem to introduce another factor on top of that. This is especially relevant because the Purcell structure suppresses the circular microwave field, which naturally raises the question of whether future improvements might require placing a microwave source inside the structure rather than driving from outside.

The supplementary material states that the total preparation time is around 400 us, which is not negligible compared with a 10 ms lifetime. However, I do not see a clear statement in the manuscript of the overall state preparation fidelity or of the protocol used to evaluate it. From Fig. 3c, the MCP counting signal appears to be already below 0.5 even at zero holding time. This again connects back to the broader claims regarding improved quantum information processing: if the state preparation fidelity is already limited, then the long lifetime alone may not directly improve the overall performance.

I therefore think the manuscript would benefit from a more detailed discussion of the preparation fidelity. In particular, the authors should clarify how the fidelity is defined and measured in the present setup, and what contributions come from the initial low-l preparation, the Landau-Zener transfer, the Purcell environment, and the detection process. Even if a full fidelity budget is difficult to provide, a discussion of the dominant limitations and possible routes toward improvement would be very valuable. For example, the authors could comment on whether optimized pulse shaping, improved microwave delivery, or a modified field geometry might increase the total preparation fidelity in future work.

Minor comment

A minor point is that, in the discussion of “neutral atom tweezer experiment, instead of cryogenic cooling [24, 25],” it would be useful to also mention the recent paper from the Princeton group (arXiv:2602.05959), which seems relevant to this context.

Reviewer #2

(Remarks to the Author)
The manuscript by Pultinevicius and co-authors describes the creation and characterization of circular Rydberg atoms with very large principal quantum number (n=103) and long lifetimes. Circular Rydberg atoms are interesting because their high angular momentum prevents radiative decay to low-lying states, allowing longer lifetimes than low-L states. Since the interaction strengths are comparable to low-L Rydberg states, this could enable more coherent quantum simulation or computing.
While this manuscript reports record-breaking results for circular Rydberg state lifetime and quantum number, my main concern is that the techniques appear to be virtually indistinguishable from a prior work by the same authors in Ref. 34, where trapping of circular Rydberg atoms with n=79 and a lifetime of 2.5 ms was achieved. Unless there was some conceptual or technical advance that enabled going to n=103 in this work, I am not sure that the additional factor of four increase in lifetime is a sufficiently impactful result by itself to merit publication in Nature Communications.
I have several minor comments as well:
1. Previous Nobel prizes are invoked several times in in the introduction as a way of arguing that a topic is interesting. This is an unusual form of argument for a scientific publication.
2. (line 58) The cavity is described as providing conditions akin to a 14 K environment. I don’t think it’s accurate to describe it that way – the cavity does not suppress pi-polarized transitions (so at best it’s some kind of anistropic temperature?). Moreover, the most striking feature of low temperature would be an asymmetry between absorption and emission rates, and that is not present here – an atom is basically as likely to transition up to a higher level as it is to transition down, because kT >> spacing between energy levels. This is clearly observed in Fig. 3 c,d. The state lifetime is what would be observed in a 14K environment, but nothing about the radiative environment itself could be said to resemble 14 K.
3. (line 71) “essentially dissipationless” is not sufficiently precise.
4. (line 19) “We observe tweezer trapping on the few hundred ms scale” – this is not consistent with the results, which show a 130 ms trapping timescale.

Version 1:

Reviewer comments:

Reviewer #1

(Remarks to the Author)
The revised manuscript has addressed most of my previous concerns, and the overall revision is satisfactory. In particular,

the authors have substantially narrowed the discussion of potential applications, focusing primarily on quantum simulation, and now present the measurement of the coherence time (T2) of the long-lived, high-(n) circular Rydberg states as an important direction for future work. This provides a more appropriately qualified perspective on the implications of the demonstrated long (T1).

The authors have also added a discussion of the fidelity of the Landau–Zener transfer process. The average population-transfer fidelity for each individual step is approximately 94%, which, when accumulated over the full sequence, results in an overall preparation fidelity of approximately 50% for the final circular Rydberg state. The manuscript also discusses possible future improvements through pulse engineering and hardware upgrades. These additions adequately address my previous request for a clearer discussion of the state-preparation efficiency and its present limitations.

I have one remaining concern regarding the treatment and presentation of the population data stated in the Supplementary Information. The authors state:
“The populations of the respective states are determined from the detected ion counts in all accessible bins with the used ionization ramp. Consequently, the initial target circular state population for the lower CRS branch (Fig. 3(c)) does include the 70% fidelity of CRS preparation [30], while the higher CRS branch (Fig. 3(d)) does not.”

I do not understand how the word “consequently” connects the preceding description of the ion-count analysis to the decision to include the initial 70% circular-state preparation fidelity in Fig. 3(c), but not in Fig. 3(d). More importantly, the two datasets appear to be normalized or processed under different conventions. This makes a direct comparison between the lower and higher circular-Rydberg-state branches potentially misleading.

In my view, the data in Figs. 3(c) and 3(d) should be presented using the same normalization and state-preparation convention. If Fig. 3(c) includes the finite initial circular-state preparation fidelity, then Fig. 3(d) should also include the corresponding preparation losses. Under such a consistent treatment, the reported population of the higher-(n) circular Rydberg states may be lower, but it would more accurately reflect the total experimental preparation efficiency.

I strongly encourage the authors to revise the analysis or presentation of Figs. 3(c) and 3(d) so that both datasets are treated consistently, and to clarify precisely which preparation and detection efficiencies are included in the reported populations.

Apart from this remaining issue, I am satisfied with the revision. As stated in my previous report, I believe that the manuscript presents a significant experimental achievement and is suitable for publication in Nature Communications once the population normalization and comparison discussed above are clarified.

Reviewer #2

(Remarks to the Author)
I thank the authors for taking the time to consider my questions. I am satisfied with the responses and the clarification of the novel aspects relative to prior works. In particular, I find the mapping of the LDOS suppression deep within the cutoff of the capacitor interesting and novel.

FWIW, I still find the invocation of the Nobel prize to be off-key for a serious publication--you shouldn't need to borrow the Nobel committee's work to argue why something is interesting.

Open Access This Peer Review File is licensed under a Creative Commons Attribution 4.0 International License, which permits use, sharing, adaptation, distribution and reproduction in any medium or format, as long as you give appropriate credit to the original author(s) and the source, provide a link to the Creative Commons license, and indicate if changes were made.
In cases where reviewers are anonymous, credit should be given to 'Anonymous Referee' and the source.
The images or other third party material in this Peer Review File are included in the article's Creative Commons license, unless indicated otherwise in a credit line to the material. If material is not included in the article's Creative Commons license and your intended use is not permitted by statutory regulation or exceeds the permitted use, you will need to obtain permission directly from the copyright holder.
To view a copy of this license, visit https://creativecommons.org/licenses/by/4.0/

5th Institute of Physics

Dr. Florian Meinert

01.07.2026

Dear Editors of Nature Communications,

We hereby resubmit our manuscript entitled "**Long-lived giant circular Rydberg atoms at room temperature**". Let us first thank the two referees for their positive assessment of our work and their constructive comments and questions, which helped us to improve the manuscript. We address them in the following point-by-point. All changes made in reply to the reviewers' questions are marked in red in the revised manuscript.

Reply to Referee #1:

Referee #1*:* *The manuscript under consideration investigates long-lived giant circular Rydberg atoms at room temperature. The two principal results are: (1) coherent control of individual circular Rydberg levels up to n = 103 with lifetimes exceeding 10 ms, and (2) optical tweezer trapping of Rydberg atoms on the ~100 ms timescale.*

These are very impressive achievements. In particular, the work is a natural and important extension of the authors' previous paper, PHYSICAL REVIEW X 14, 021024 (2024), in which they demonstrated the generation of n = 79 circular states from low-l states. The present manuscript substantially extends this capability to higher principal quantum numbers and provides a systematic study of the lifetime enhancement for different circular states. The observed strong suppression of spontaneous emission, attributed to the Purcell effect induced by the ITO-coated windows, appears in very good agreement with the COMSOL simulation of the full structure. The demonstration of lifetimes exceeding 10 ms is, to my knowledge, the longest-lived Rydberg state experimentally demonstrated in a room-temperature setup. This is a noteworthy and significant result for the field.

Overall, the manuscript is clearly written, well structured, and experimentally thorough. I believe the work is suitable for publication in Nature Communications. However, before acceptance, I think a few important claims would benefit from clarification and further support.

Reply*:* We thank the reviewer for the positive response and the recommendation for publication in Nature Communications.

Referee #1*:* *Major comments*

1. Clarification of claims regarding quantum information processing and simulation

In the third paragraph of the Introduction, the authors state:

"for comparison, our high n brings about a thousandfold stronger van der Waals blockade, and more than ten times larger dipole-exchange coupling. Combining these results forms the basis for establishing essentially dissipationless quantum simulation on the Rydberg platform on timescales two orders of magnitude longer than what is currently possible."

This statement feels somewhat too strong in its current form and may benefit from a more careful discussion.

A longer T1 is certainly helpful, especially in Rydberg-based platforms. However, it is not yet clear that the strong blockade of circular Rydberg states can be directly exploited in a practical gate protocol, since excitation first starts from low-l states and then requires an adiabatic RF or microwave transfer to the final circular state. As far as I know, there is no direct blockade-gate scheme that uses the circular-state blockade in the same way as standard low-l Rydberg gates.

In fact, previous proposals such as PRX Quantum 2, 030322 (2021) implement gates through coherent Sz_i Sz_j-type interactions, where the relevant figure of merit is not only T1, but also T2. Similarly, for quantum simulation applications, the coherence time can be limited by several other effects, including atomic motion and electric-field stability. The latter may become even more important at higher n because of the stronger polarizability of the circular states. In addition, for circular states, interaction-induced atomic motion has recently been discussed in PRX Quantum 6, 010353 (2025), which suggests that motional effects may also become relevant in the coherent dynamics.

For these reasons, the demonstrated enhancement of T1 does not necessarily imply a proportional improvement in coherent many-body operations or gate quality. I think the manuscript would be stronger if the authors could either soften this claim or provide additional discussion to better justify it. For example, it would be helpful to comment on the expected T2 limitations in the present platform, or to outline what technical developments would be needed for the long T1 to translate into an actual quantum information or quantum simulation advantage. A short discussion of possible future improvements, such as better electric-field control, reduced motional effects, or protocols specifically designed to exploit the circular-state manifold, would make this part much more convincing.

Reply*:*

We thank the referee for pointing this out and agree that the manuscript has so far been short in discussing the role of the T_2 time of the circular qubit. This is because the central focus of this work is the demonstration of boosting the circular state lifetime as the *fundamental limit* when using circular states for quantum simulation. To clarify this, we now comment the role of the T_2 time in the introduction, and discuss how to improve current technical limits on it in the conclusion (including the additional references mentioned below).

Specifically, as pointed out by the referee, the T_2 time is indeed the second important aspect in the path towards quantum simulation. Currently, the

qubit's T_2 coherence time reaches up to 280 us (measured for the lowest n=77 to n=79 transition we explore here via spin echo, see our previous work [34]) is limited by technical imperfections which can be improved on. Those are merely static electric field gradients and temporal magnetic field fluctuations. Magnetic field noise can, for instance, be reduced by established feedback techniques which we expect to reach an order of magnitude improvement. An in depth characterization of the electric potential landscape around the tweezer array should provide the means for compensation of aforementioned electric field gradients. With progressing work on addressing of the ionic core, we expect to be able to motionally ground state cool the circular states after their preparation in order to reduce motion induced decoherence (see Bouillon et al., PRL **132**, 193402 (2024)).

With all of these steps in mind, which are all on our agenda, we are optimistic that coherence times on the millisecond scale are within reach. Finally, more options, such as dynamical decoupling sequences, can be explored to further improve on coherence times throughout quantum simulations. We believe that decoupling techniques similar to what has been recently shown for interacting dipolar molecules (see Holland et al., Science **382**, 6675 (2023) and related recent theory work Choi et al., PRX **10**, 031002 (2020)) can be also adapted to our circular state platform. This is particularly true for static electric gradient fields and motional dephasing, as microwave based dynamical decoupling pulses (<1µs) are much faster than the typical timescales of motion in the tweezer (~10…100 kHz trap frequency).

Referee #1*:* *2. State preparation fidelity should be discussed more clearly*

I also think it is important to state the fidelity of the Landau-Zener preparation process more explicitly.

*In the previous paper (Hölzl et al., PRX **14**, 021024 (2024)), the fidelity to* **|***79C> was already around the 70% level. In the present work, the current Landau-Zener process to reach higher n would seem to introduce another factor on top of that. This is especially relevant because the Purcell structure suppresses the circular microwave field, which naturally raises the question of whether future improvements might require placing a microwave source inside the structure rather than driving from outside.*

The supplementary material states that the total preparation time is around 400 us, which is not negligible compared with a 10 ms lifetime. However, I do not see a clear statement in the manuscript of the overall state preparation fidelity or of the protocol used to evaluate it. From Fig. 3c, the MCP counting signal appears to be already below 0.5 even at zero holding time. This again connects back to the broader claims regarding improved quantum information processing: if the state preparation fidelity is already limited, then the long lifetime alone may not directly improve the overall performance.

I therefore think the manuscript would benefit from a more detailed discussion of the preparation fidelity. In particular, the authors should clarify how the fidelity is defined and measured in the present setup, and what contributions come from the initial low-l preparation, the Landau-Zener transfer, the Purcell environment, and the detection process. Even if a full fidelity budget is difficult to provide, a discussion of the dominant limitations and possible routes toward improvement would be very valuable. For example, the authors could comment on whether optimized pulse shaping, improved microwave delivery, or a modified field geometry might increase the total preparation fidelity in future work.

Reply*:* We thank the referee for this remark and agree that the manuscript would benefit from a more detailed description of the novel Landau-Zener (LZ) transfer. Accordingly, we have added two new paragraphs to Appendix A and B.

Briefly, for each n manifold, the population is determined by taking all bins into account which are detected with the used SSFI parameters (see Appendix B). Generally, the populations of the initialized circular state at t_h=0 are subject to the following contributions:

1.) The preparation fidelity of the initial circular state (n=79). This is similar to what we already reported in our previous PRX (Hölzl et al., PRX **14**, 021024 (2024)), namely 70%. We give this number now in the new paragraph of Appendix B.

2.) The fidelity of the Landau-Zener sweeps. This varies depending on the specific transition. We find an average population transfer fidelity between circular states n → n+2 of ~94%. This value is obtained from the initial histograms of the lifetime measurements used in Fig. 3. We give this number now in Appendix A.

These contributions explain the initial state populations of the datasets shown in Fig.3(c) and (d). For example, the 5 LZ ramps needed to populate the initial state n=89 (Fig.3(c)) and the initial n=79 CRS preparation efficiency combine to ~0.5. Note that for the higher-lying CRS populations contribution one does not apply, because the n=79 manifold is not detected in the used SSFI (see Appendix B).

For each individual LZ transition we have adapted microwave power, sweep time, and center frequency to reach the above mentioned typical transfer efficiency, but have not extensively optimized all the steps. The population transfers could, for example, be further increased by parametrization of the voltage ramp or shaping of the MW pulses. We have not seen fundamental limitations from the fact that the microwave is coupled in from outside the capacitor structure. Indeed one could likely shorten the sweep times by adding additional MW amplifiers. Though for some transitions one has to be careful that disturbing single-photon transitions are sufficiently far off-resonant (see Appendix A, Fig. 5). Such resonances appear because of the presence of all polarization modes being supported (and potentially even enhanced) by the capacitor. A better polarization control for the microwave, e.g. by integrating high-bandwidth antenna structures into the electric field control) is nevertheless an interesting aspect for a second-generation experiment.

Furthermore, the total transfer time from n=79 to n=101 currently adds up to 410µs. A significant part of it (220 µs in total, 20 us per pulse) is swichting time of the microwave generator between frequencies. Note that meanwhile, we were able to reduce this to 3us (per pulse), both by firmware improvements and combination of two phase-coherent microwave sources. This already greatly reduces the total preparation time by 187µs to only ~2% of the longest measured circular state lifetime in all future experiments.

Finally, we want to emphasize that the purpose of this particular transfer over a range of more than 20 n is the characterization of the blackbody suppression in our electric field control. For quantum simulations we would directly prepare the initial CRS via the adiabatic rapid passage, after deciding the target qubit states.

Referee #1*:* *Minor comment*

A minor point is that, in the discussion of "neutral atom tweezer experiment, instead of cryogenic cooling [24, 25]," it would be useful to also mention the recent paper from the Princeton group (arXiv:2602.05959), which seems relevant to this context.

Reply*:* We thank the referee for pointing out the recent cryo-paper by the Princeton groups. We have added the reference accordingly as the new Reference [26].

Reply to Referee #2:

Referee #2*:* *The manuscript by Pultinevicius and co-authors describes the creation and characterization of circular Rydberg atoms with very large principal quantum number (n=103) and long lifetimes. Circular Rydberg atoms are interesting because their high angular momentum prevents radiative decay to low-lying states, allowing longer lifetimes than low-L states. Since the interaction strengths are comparable to low-L Rydberg states, this could enable more coherent quantum simulation or computing.*

While this manuscript reports record-breaking results for circular Rydberg state lifetime and quantum number, my main concern is that the techniques appear to be virtually indistinguishable from a prior work by the same authors in Ref. 34, where trapping of circular Rydberg atoms with n=79 and a lifetime of 2.5 ms was achieved. Unless there was some conceptual or technical advance that enabled going to n=103 in this work, I am not sure that the additional factor of four increase in lifetime is a sufficiently impactful result by itself to merit publication in Nature Communications.

Reply*:* We thank the reviewer for the assessment of our work. In the following, we want to clear up the referee's concerns about the novelty of this work as compared to our previous PRX (Ref. [34]). The central technical advances and the new measurements reported in the present paper are:

1.) We report for the first time a full characterization and an in-depth understanding of the Purcell suppression of black-body radiation in the capacitor structure. Importantly, our previous experiment [34] was very limited in principal quantum numbers to n=77 and n=79 only. Consequently, we could only observe the very onset of Purcell suppression, and a detailed understanding of what lifetimes can be achieved in a room temperature setup was not possible.

Now, by realizing access to much higher values of *n*, we managed to enter into the deep black-body suppression regime, where the half-wavelength of the dominating black-body transition exceeds the capacitor gap. This not only gave rise to the reported increase in lifetime (>10ms around *n*=100), but also revealed the importance of additional resonances of π-polarized radiation modes stemming from the 3D electrode structure, and the role of transitions to next-neighboring manifolds. The combination of all these findings is of importance for selecting ideal states for future quantum simulations. Our work shows, that the details of the capacitor structure are of central importance when designing black-body suppression structures for Rydberg states. The measurement data and the Purcell suppression are also supported for the first time by an in-depth numerical analysis revealing the relevance of the mentioned effects.

2.) The present manuscript reports for the first time access to circular states covering a wide range of record-high principal quantum numbers (n=77 to

n=103). This constitutes the largest circular states (1.1 µm diameter) produced to date. As already mentioned above, in our previous work, we could only access n=77 and n=79. Key to large circular states was a major upgrade of the microwave infrastructure on our machine, which allows now for rapid frequency switching in the 6…13 GHz range. This enabled the implementation of the circular state LZ ladder on timescales faster than typical Rydberg lifetimes, by chaining up to 11 microwave pulses spanning a range of more than 30 Rydberg manifolds.

3.) We report for the first time trapping times of circular states in access of 100ms (as a lower bound). The longest hold times in the tweezers we could observe in our previous paper was on the order of 1 ms (limited by technical constraints of our electric field control electronics), which did not allow us to infer trapping times. The very long trapping times we observe now open interesting perspectives not only for quantum simulation, but also high-resolution metrology on the inner core transition, and coherent control of motion of Rydberg atoms in the tweezers (as we state in the conclusion).

We hope that these points clearly establish the novelty and added value of the present manuscript relative to our previous work, and that they will convince the referee that it is suitable for publication in Nature Communications.

We emphasize the characterization of the Purcell structure by adding a paragraph to the introduction and conclusion each:

Introduction: *"In our finite size setup, with further electrodes and conductive elements (see Fig. 1(a)), deviations from this idealized model occur due to formation of additional resonant modes. Here, we characterize these effects on the BBR environment by going far beyond the onset of Purcell suppression at $\lambda \approx 2d$."*

Conclusion: *"We characterized the Purcell effect on black-body radiation far beyond the cut-off condition of our suppression structure via the enhancement on circular state lifetimes over a wide range of coherently connected n. This revealed the effects of additional resonant modes inside our electric field control in agreement with our theoretical models, demonstrating important aspects in the design of black-body modification structures."*

Referee #2*:* *I have several minor comments as well:*

1. Previous Nobel prizes are invoked several times in in the introduction as a way of arguing that a topic is interesting. This is an unusual form of argument for a scientific publication.

Reply*:* We thank the referee for his feedback. We still believe that it is useful to point out the Nobel prize work by the Haroche group, but agree that it is sufficient to state this only once (in the introduction).

We rephrase in the abstract "Nobel prize-winning" to "seminal".

Referee #2*:* 2. (line 58) The cavity is described as providing conditions akin to a 14 K environment. I don't think it's accurate to describe it that way – the cavity does not suppress pi-polarized transitions (so at best it's some kind of anistropic temperature?). Moreover, the most striking feature of low temperature would be an asymmetry between absorption and emission rates, and that is not present here – an atom is basically as likely to transition up to a higher level as it is to transition down, because kT >> spacing between energy levels. This is clearly observed in Fig. 3 c,d. The state lifetime is what would be observed in a 14K

environment, but nothing about the radiative environment itself could be said to resemble 14 K.

Reply*:* We are grateful for this constructive feedback. We are aware that a modified black-body spectrum as realized in our capacitor is not described by a thermal distribution of photons in free space. Indeed, our intention was to compare only one specific metric, the lifetime (in the range of n where the 10 ms range is reached), to what would be needed in a cryogenic temperature environment to achieve the same results. To avoid confusion, we have rephrased the corresponding sentence and now write:

"… effectively provides lifetimes that in free space would require a 14 K environment."

Referee #2*:* 3. (line 71) "essentially dissipationless" is not sufficiently precise.

Reply*:* The referee is right that we have not been very precise here, and now omit "essentially dissipationless". What we wanted to emphasize is that these long lifetimes allow for long quantum simulation times in the absence of state decay. The sentence now reads:

In accordance with the questions raised by referee 1, we have now modified the paragrah:

"Under the condition that the technical factors limiting the currently achieved dephasing time are addressed, these results push the fundamental barrier (i.e. the Rydberg state's T_1 time) for establishing quantum simulation on the Rydberg platform on timescales two orders of magnitude longer than what is currently possible."

Referee #2*:* 4. (line 19) "We observe tweezer trapping on the few hundred ms scale" – this is not consistent with the results, which show a 130 ms trapping timescale.

Reply*:* We thank the referee for this remark, and rephrase the statement more accurately. We now write in the abstract:

"We … observe tweezer trapping of the Rydberg atoms on the hundred millisecond scale."

Additional minor changes to the manuscript:

- Added subsection numbering (A, B, C, ...) to methods to fix references to the Appendix.
- The LZ ladder up to n=101 takes ~ 400µs, not up to n=103
- Introduction of LZ short for "Landau-Zener" in the main text.

To conclude, we think that the manuscript in its improved form now meets the standards of Nature Communications and are confident that it can be published now.

Yours faithfully,

Florian Meinert (on behalf of all authors)

5th Institute of Physics

Dr. Florian Meinert

07.08.2026

Dear Editors of Nature Communications,

We hereby resubmit our manuscript entitled "**Long-lived giant circular Rydberg atoms at room temperature**". Let us first thank the two referees for their positive assessment of our work and their recommendation for publication. Below, we answer the remaining comment by referee 1. We also considered it in the revised manuscript as detailed in our reply. We hope that our reply obviates the need for further review.

Reply to Referee #1:

Referee*: I have one remaining concern regarding the treatment and presentation of the population data stated in the Supplementary Information. The authors state:*

"The populations of the respective states are determined from the detected ion counts in all accessible bins with the used ionization ramp. Consequently, the initial target circular state population for the lower CRS branch (Fig. 3(c)) does include the 70% fidelity of CRS preparation [30], while the higher CRS branch (Fig. 3(d)) does not."

I do not understand how the word "consequently" connects the preceding description of the ion-count analysis to the decision to include the initial 70% circular-state preparation fidelity in Fig. 3(c), but not in Fig. 3(d). More importantly, the two datasets appear to be normalized or processed under different conventions. This makes a direct comparison between the lower and higher circular-Rydberg-state branches potentially misleading.

In my view, the data in Figs. 3(c) and 3(d) should be presented using the same normalization and state-preparation convention. If Fig. 3(c) includes the finite initial circular-state preparation fidelity, then Fig. 3(d) should also include the corresponding preparation losses. Under such a consistent treatment, the reported population of the higher-(n) circular Rydberg states may be lower, but it would more accurately reflect the total experimental preparation efficiency.

I strongly encourage the authors to revise the analysis or presentation of Figs. 3(c) and 3(d) so that both datasets are treated consistently, and to clarify

precisely which preparation and detection efficiencies are included in the reported populations.

Reply: As pointed out in the previous reply, the populations are analyzed with the same procedure in all measurements. The 70% circular state preparation fidelity is not included by artificial scaling of the populations, but is inherently included in the evaluation. The remaining 30% are residual elliptical states which are all detected in the same bin as the n=79 CRS and cannot be distinguished by our SSFI (see also [30]). These are not addressed by our microwave pulses and mostly remain in the same bin, as can also be seen in Fig. 1(d). Since the detection range for all higher-lying CRS starts at n=89, the remaining elliptical states from the circular state preparation are not detected and consequently not included.

Finally, we want to point out that we require normalized populations across the detected bins for the rate-equation model which is fitted to extract the lifetimes. For that reason, we do not scale the populations as shown in Fig. 3(d) to include the preparation efficiency.

In our manuscript, we now extend our statement in the methods section to make this more clear:

„The populations of the respective states are determined from the detected ion counts in all accessible bins with the used ionization ramp. This includes residual elliptical states in the n=79 bin, which are not addressed by our MW pulses (see also Fig. 1(d)). Consequently, the initial target circular state populations of n > 79 for the lower CRS branch (Fig. 3(c)) do include the 70% fidelity of CRS preparation [30], while the higher CRS branch (Fig. 3(d)) does not.“

Yours faithfully,

Florian Meinert (on behalf of all authors)
